# Relationship between Physical Performance, Anthropometric Measurements and Stroke Velocity in Youth Tennis Players

**DOI:** 10.3390/sports11010007

**Published:** 2022-12-28

**Authors:** Koulla Parpa, Marcos Michaelides, Dennis Petrov, Christos Kyrillou, Ana C. Paludo

**Affiliations:** 1 Faculty of Sports and Exercise Science, UCLan University of Cyprus, Pyla 7080, Cyprus; 2 Department of Kinesiology and Health Education, University of Texas, Austin, TX 78712, USA; 3 Faculty of Sports Studies, Incubator of Kinanthropology Research, Masaryk University, 625-00 Brno, Czech Republic

**Keywords:** tennis, athletic performance, physical fitness, youth athlete

## Abstract

Given that serve velocity has been identified as one of the most important components influencing performance in tennis, identifying the factors associated with serve velocity is crucial for coaches and athletes. The aim of this study was to describe the relationship between physical performance, anthropometric characteristics and stroke velocity in youth tennis players. Twenty-seven youth players (male = 16, age = 15.69 ± 1.70 years; female = 11, age = 15.82 ± 1.40 years) underwent an anthropometric and physical performance assessment. On a tennis court, players were assessed for forehand, backhand and serve velocities. Pearson’s correlation coefficient revealed that forehand velocity was significantly correlated with height (r = 0.58) and handgrip strength (right hand: r = 0.68; left hand: r = 0.57), whereas backhand velocity was significantly correlated with running time (r = 0.52) and handgrip strength (right hand: r = 0.67; left hand: r = 0.55) in males. Similarly, in males, serve velocity was significantly correlated with height (r = 0.60), running time (r = 0.62) and handgrip strength (right: r = 0.77, left hand: r = 0.71). In females, a significant correlation was only demonstrated between serve velocity and body weight (r = 0.69). These findings highlight that handgrip strength, running time and body height variables are positively associated with stroke velocities in male youth tennis players.

## 1. Introduction

In contemporary tennis, predicting variables involved in players’ performance could help coaches and physical trainers design an effective training plan in order to enhance sport-specific performance. It is well-established that tennis matches require strength, cardiovascular endurance, speed and power to perform high stroke velocities, together with a complex interaction of technical and tactical skills [1,2,3,4,5,6,7]. Therefore, it is possible to infer that one predominant factor cannot define a successful performance. 

Evidence supports that among the factors involved in tennis matches, the velocity of strokes (forehand, backhand and service) is an important element with respect to the success of the match outcome [8,9]. Thus, improving a player’s stroke efficiency is of interest to those involved with tennis players’ performance, especially sports scientists. A previous study investigated the functional performance and anthropometric factors associated with junior tennis players’ service velocity. It was the first study that used a regression analysis to combine the factors, and the authors concluded that some selected physical components, such as upper-body strength, power and anthropometric parameters (body height, arm span and body mass), play a significant role in youth tennis players’ service speed [4]. 

Indeed, physical and anthropometric parameters have been demonstrated to influence service velocity; however, there is no consensus about which components and measurements can be used as good performance predictors. Nevertheless, targeting these components can be valuable for youth players in the phase of their specialization in a tennis discipline, as well as for talent identification programs [4]. Therefore, the aim of this study was to examine the relationship between physical performance and anthropometric characteristics with forehand, backhand and service velocity in youth tennis players. Based on previous research [4], we hypothesize that strength, power and anthropometric components are significantly correlated with forehand, backhand and service velocities. 

## 2. Materials and Methods

### 2.1. Participants 

A total of 27 youth tennis players (male = 16, age: 15.69 ± 1.70 years; female = 11, age: 15.82 ± 1.40 years) belonging to the same tennis academy volunteered to participate in the study. All players engaged in formal training sessions (3–4 sessions/week, ~90 min/session) and participated in at least two competitive seasons. Furthermore, all the players reported to be ‘right-handed’. Players’ characteristics are displayed in Table 1. The exclusion criteria included injuries or sickness resulting in losing training sessions two months prior to study initiation. Parents or legal guardians signed an informed consent after being briefed about the study’s risks, procedures and benefits. Additionally, players received medical clearance to participate in tennis training and testing. The study was performed in accordance with the Declaration of Helsinki and was approved by the Ethics Committee of the University (reference number STEMH 541) and the National Committee on Bioethics [10].

### 2.2. Study Overview

**Figure 1:**
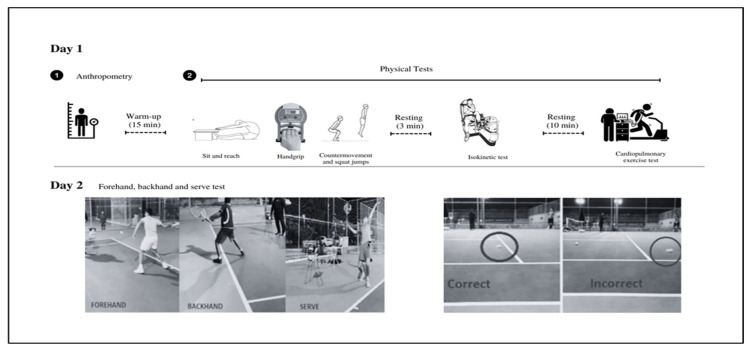
Study design.

Players were assessed on two days during the off-season period (Figure 1). The measurements were obtained between 9:00 a.m. and 14:00 on two different days to avoid potential fatigue from subsequent testing measurements. On the first day, players underwent an anthropometric evaluation (body height and body composition) followed by a physical performance assessment (sit and reach, hand grip, countermovement and squat jumps, and isokinetic and cardiopulmonary testing). On the following day, players performed specific tests (forehand, backhand and serve) on a tennis court. A familiarization period as provided before the evaluations, as some tests were not part of the athletes’ usual training routine. The players were advised to abstain from any activity in the days before testing.

### 2.3. Anthropometric Evaluation 

Anthropometric measurements included body height and body mass. Stature and body mass were measured according to standard procedures [11] using a wall stadiometer (Leicester; Tanita, Tokyo, Japan) and a standard electronic scale and recorded to the nearest 0.1 kg and 0.1 cm, respectively. Body fat was measured with a bioelectrical impedance analyzer (BC418MA; Tanita). 

### 2.4. Physical Tests

Physical performance tests were conducted in the order presented below. The recovery time between the performance tests was set to at least 3 min.

Sit and Reach Test: a custom sit and reach box (32.4 cm high and 53.3 cm long) with a 26 cm heel line mark was used to assess the flexibility of the lower back and hamstring muscles according to methods described by previous investigators [12]. Players placed the soles of their feet (no shoes) against the box with their knees in full extension. They were instructed to lean forward with one hand on top of the other and palms facing downward. Fast and jerky movements were not allowed while they were leaning forward. Three attempts were performed, and the best trial recorded to the nearest centimeter was retained. 

Handgrip Strength Test: A handgrip dynamometer (Takei Scientific Instruments Co., Ltd., Tokyo, Japan) was used to assess the maximum isometric strength of the forearm and hand muscles. The procedure was conducted according to the methods described by previous investigators [13]. Two attempts were performed, and the highest value was retained. 

Countermovement Jump (CMJ) and Squat Jump (SJ): Explosive strength power was assessed with CMJ and SJ tests. Vertical jump performance (CMJ and SJ) was evaluated using OptojumpTM photoelectric cells (Microgate, Bolzano, Italy) based on previously described methods [14]. Each player performed three CMJs and three SJs with the same break between jumps. They were instructed to stand between Optojump bars, and the first task was to perform an SJ with their knee joint bent approximately 100 degrees. The tennis player had to descend into a semi-squat position and hold that position for approximately 3 seconds before takeoff. The task was repeated three times at 15 second intervals, and the maximum of the three trials was recorded for statistical analysis. Thereafter, each tennis player performed three consecutive CMJs with the same break between jumps. In the CMJ, the players started from a standing position and initiated a downward movement, followed by an upward movement leading to takeoff. In both cases, the participant’s hands were placed on their waist, and swinging of the arms was not allowed. The highest of the three valid jumps was included in the data analysis. 

Isokinetic strength measurements: Isokinetic knee strength was assessed utilizing a Humac Norm and Rehabilitation device (CSMI, Stoughton, MA, USA) according to the methodology described by previous investigators [15]. Before isokinetic testing, tennis players had a 5 min warmup on a mechanically braked cycle ergometer (Monark 894 E Peak Bike, Weight Ergometer, Sweden). Once the players were appropriately positioned on the device, they performed five sub-maximal repetitions of concentric knee flexion and extension for familiarization purposes. The isokinetic testing included three maximal concentric flexion and extension repetitions at an angle speed of 60°/s. The maximum torque (Nm) out of the three repetitions was retained for analysis from both quadriceps and hamstring muscles. 

Cardiopulmonary exercise testing: The players completed incremental maximal cardiopulmonary exercise testing until they reached exhaustion on a treadmill (h/p/Cosmos Quasar med, H-P-Cosmos Sports and Medical GmbH, Nussdorf-Traunstein, Germany). The players were tested utilizing the modified Heck incremental maximal protocol described by previous investigators [16,17]. A breath-by-breath analysis was performed on a Cosmed Quark CPET (Rome, Italy) system, and laboratory conditions were kept constant (temperature, 22 ± 1 °C; relative humidity, 50%). The test was terminated once the participant reached volitional fatigue or when there was no variation among the maximal oxygen consumption (VO2max) levels as the workload increased. The VO2max was detected after filtering the results to identify the highest value for an average of 10 seconds. The ventilatory (VT) and respiratory compensation points (RC) were determined using different criteria. The ventilatory threshold was determined through the V-slope method and was verified at the nadir of the VE/V O2 curve. The respiratory compensation point was determined at the nadir of the VE/V CO2 curve.

### 2.5. On-Court Stroke Tests (Forehand, Backhand and Serve)

On-court performance was measured on a different day on an outdoor standard acrylic tennis court. The tennis players were provided with a dynamic warmup and appropriate time for familiarization with the assessment procedure. Two cameras (Sony AX33 Handycams filming at approximately 8.28 megapixels) were set up during the testing based on procedures described by previous investigators [18]. One camera recorded the player’s stroke execution, and the other camera recorded the balls landing after the stroke. The recording was completed in normal-speed and slow-speed modes. Attempts that did not land within game-regulated areas were not included in the data analysis. The ball’s top speed was measured for the three major tennis strokes (serve, forehand and backhand) using a radar gun (SR 3800 long-range speed radar gun). The radar was positioned and calibrated based on the manual’s instructions. The radar was centered 1.5 meters on the posterior side of the player, facing the ball’s trajectory. In addition, the radar was elevated two meters off the ground and provided data on a numeric screen with the execution of each stroke. All participants were required to perform five strokes using the three techniques under the supervision of two International Tennis Federation-certified tennis coaches. The participants were required to perform as many shots as possible until they achieved a total of five correct executions (within the right/left service area) from each stroke technique. All tennis balls (Wilson Regular use) were brand-new and used at the professional level of tennis. The highest velocity out of the five correct executions from each stroke was recorded for the statistical analysis.

### 2.6. Statistical Analysis 

The homogeneity of variance and normality assumptions were verified using Brown and Forsythe’ and Shapiro–Wilk’s tests, respectively. All parameters are presented as mean and standard deviations, as the normality was confirmed. Pearson-product moment correlation coefficients were used to determine the contribution of physical performance and anthropometric measures in on-court tennis tests (forehand, backhand and serve velocity). Correlations were referred to as trivial (0–0.1), small (0.1–0.3), moderate (0.3–0.5), large (0.5–0.7), very large (0.7–0.9), nearly perfect (0.9) and perfect (1.0) [19]. Male and female players were compared using the independent samples t-test. Cohen’s d was calculated to determine the effect size (ES) to present the magnitude of the reported effects. ES was interpreted as small (0.2–0.4), medium (0.5–0.7) and large (0.8–1.4) [20]. All statistical analyses were performed in IBM^®^ SPSS^®^ Statistics, version 26.0, for Windows (SPSS Inc., Chicago, IL, USA ), and significance was set at *p* < 0.05. 

## 3. Results

Players’ anthropometric and body composition measurements are presented in Table 1. Female players presented significantly lower body height and mass and higher body fat compared to male players. 

Table 2 describes the physical performance and tennis stroke velocity measurements. Regarding physical performance, results indicated that the male players presented a significantly better performance in the right (t(25) = 5.15, d = 2.11, *p* < 0.05) and left handgrip strength (t(25) = 5.84, d = 2.31, *p* < 0.05), as well as countermovement jump performance (t(25 )= 6.78, d = 2.70, *p* < 0.05), compared to the female players. Male players also demonstrated better cardiorespiratory performance compared to the female players, as indicated by running time (t(25) = 6.71, d = 2.67, *p* < 0.05), VO2max (t(25) = 3.50, d = 1.47, *p* < 0.05), vRC (t(25) = 4.10, d = 1.51, *p* < 0.05) and _V_VO2max (t(25) = 6.61, d = 2.66, *p* < 0.05). For court tests, significant differences were demonstrated in the male and female tennis players’ forehand (t(25)= 3.75, d = 1.38, *p* < 0.05), backhand (t(25) = 3.62, d = 1.47, *p* < 0.05) and serve (t(25) = 6.72, d = 2.70, *p* < 0.05) velocities, with male players presenting better results than female players.

The measurements of the isokinetic test are presented in Table 3. Male players demonstrated significantly greater relative torque production for quadriceps and hamstring muscle groups at 60 and 300 degrees/s for the right and left legs.

The correlation between anthropometric and physical performance measurements and forehand, backhand and serve ball velocities are presented in Table 4. In male players, the forehand velocity was significantly associated with height and relative right and left handgrip strength, whereas backhand velocity was significantly associated with running time and relative right and left handgrip strength. Serve velocity was significantly associated with height, running time on the treadmill and relative right and left handgrip strength. Significant correlations were classified as ‘large’. The only significant association for female players was demonstrated between serve velocity and weight.

## 4. Discussion

The aim of the present study was to examine the relationship between physical performance, anthropometric characteristics and stroke velocity of male and female tennis players. First, we found that male youth players presented better results in some physical tests (relative handgrip strength, CMJ, VO2max, running time and isokinetic measurements) and higher forehand, backhand and serve velocities compared to the female players. Regarding the relationship amongst the variables, the highest correlations (large) were demonstrated between handgrip strength and forehand, backhand and serve velocities in male youth players but not for female players. Body height is an anthropometric variable that presents a significant and large relationship with forehand and serve velocities in males. Additionally, a significant relationship was demonstrated between running time on the treadmill and serve and backhand velocities in male players. On the other hand, in female players, a significant and moderate relation was only found between body weight and serve velocities.

Regarding the serve velocity and physical and anthropometric variables, the results obtained in the present study confirm the results of a previous study, which also indicated a significant influence of upper-body strength and body height in males [4]. In our study, the correlations between serve velocity and handgrip measurement were classified as large (r = 0.63 and 0.61)—higher than those reported in a study by Fett et al. [4], in which the correlation in youth players ranged from 0.43 to 0.59. Body height in our study also had a higher correlation (r = 0.60 versus 0.31 to 0.52). Similarly, Baiget et al. [21] described that the tallest male tennis players have a better disposition to serve fast, with a large (r = 0.50) and moderate (r = 0.32 and 0.49) influence. Additionally, a study by Sánchez-Pay et al. [6] demonstrated a significant positive correlation (ranging from 0.60 to 0.93) between anthropometric measurements (body mass, height, arm, forearm and leg segments), physical parameters (hand strength and countermovement jump) and functional tests with serve velocity in national and professional level tennis players. It is reasonable to suggest that a taller person with longer body segments can have a more powerful kinetic chain. Therefore, body height may be considered an essential parameter, especially during the talent identification process. In addition to body height, handgrip strength appears to be significantly related to stroke velocities in male youth tennis players. The fact that handgrip strength is an indicator of upper-body strength emphasizes the importance of upper-body strength in tennis performance. A similar study that included only female tennis players indicated that anthropometric measures such as body weight are associated with tennis motor abilities [22]. The authors explained that higher body weight, in addition to greater body mass, might be indirectly associated with higher serve velocities, which is in agreement with our study, in which a significant and moderate relation was found between body weight and serve velocities in female players.

Regarding cardiovascular endurance, our results indicate a large significant relationship between running time on the treadmill with serve and backhand velocities in male tennis players. Although we cannot compare our treadmill results to those of other studies (as nobody used the same treadmill protocol before), the VO2max results (59.55 ± 11.46 mL/kg/min) of the male players in our study were similar to those reported by other researchers (58.5 ± 9.4 mL/kg/min) [23]. Previous research indicated that high aerobic capability is significant, as it enables the player to generate faster service, repeatedly complete rapid on-court movements and have a faster recovery [3]. Concurrently, research indicated higher physiological responses in terms of VO2, heart rate (HR), %HRmax, respiratory exchange ratio and rate of perceived exertion in serve matches compared to return matches during a one-hour simulated tennis match [24]. Considering the significant correlation between the running time on the treadmill and VO2max in our study (r = 0.6, *p* < 0.01), we can assume that running time on the treadmill may be associated with greater aerobic capacity. However, this finding should be interpreted with caution, as running time might demonstrate both the aerobic and anaerobic capability of the athletes. Therefore, it is advisable, in addition to the running time on the treadmill, to use an anaerobic performance test in order to draw more accurate conclusions. 

Interestingly, flexibility, squat jump and countermovement jump performance were not significantly related to serve, forehand or backhand velocities in our study. Regarding jump performance, our results are similar to those reported in a previously published study [6], which indicated no positive relationship between jump performance and serve speed. The authors suggested that these results may be attributed to the differences in the takeoff and landing of the players during the serve and explained that it might be more beneficial if the flight time is assessed rather than the jump height. On the contrary, Girard and Millet (2009) [25] indicated that teenage players’ squat jump and countermovement jump performance was statistically linked to specific performance levels identified via tennis ranking in a tournament. However, their study did not examine the association between those performance variables and performance during on-court movements and stroke production. 

Regarding lower-body strength, our results demonstrated no association between relative lower isokinetic strength and serve velocity, which is in agreement with some studies that indicated that lower-body isokinetic strength [26] and power [4] were not associated with serve velocity. Despite the findings mentioned above, evidence to the contrary exists. Research indicated that lower-body strength was not only associated with service speed [2,5] but that knee extension peak torque was the only variable to significantly affect ball placement [5]. 

From a practical standpoint, our results reinforce the importance of including neuromuscular strength training intervention in addition to technical training. Coaches can be advised to implement upper-body strength training programs, which was also highlighted by other researchers [27], for youth players to improve their stroke velocities. A physical test such as the handgrip strength test can also help to monitor the upper-body strength of players through the training program. Moreover, a simple anthropometric measurement, such as body height, must be considered, together with sport-specific parameters, for talent identification in youth tennis players. 

Despite the important and significant findings, this study is subject to some limitations. A major limitation is the small sample size; therefore, future studies with a larger sample size of youth players are needed to confirm the results of this study. Another limitation is that the players’ maturity status and relative age were not determined.

## 5. Conclusions

The findings of this study highlight that handgrip strength, together with running time and body height, appears to be significantly related to stroke velocities in male youth tennis players, suggesting that these factors are important to generate power production in fast strokes. Therefore, coaches should focus on those variables when developing long-term training programs and during the talent identification process.

## Figures and Tables

**Table 1:** Anthropometric and body composition characteristics (mean ± SD) of youth male and female tennis players.

Variable	Entire Group (n = 27)	Males (n = 16)	Females (n = 11)
Age (years)	15.74 ± 1.56	15.69 ± 1.70	15.82 ± 1.40
Height (cm)	172.30 ± 8.823	177.50 ± 6.06	164.73 ± 6.42 *
Weight (kg)	67.87 ± 8.95	71.06 ± 9.35	63.23 ± 6.11 *
Body mass index (kg.m^−2^)	22.90 ± 2.62	22.58 ± 2.77	23.36 ± 2.45
Body fat (%)	20.19 ± 7.50	15.00 ± 3.41	27.74 ± 4.82 *

Note: * *p* < 0.05 denotes significant differences between male and female tennis players.

**Table 2:** Performance parameters of forehand, backhand and serve velocities (mean ± SD) of youth male and female tennis players.

Variable	Entire Group (n = 27)	Males (n = 16)	Females (n = 11)	95% CI of the Difference Lower–Upper	Cohen’s *d*
Physical Test					
Relative right handgrip strength (kg/BMI)	1.88 ± 0.48	2.16 ± 0.40	1.47 ± 0.23	0.41–0.96	2.11
Relative left handgrip strength (kg/BMI)	1.63 ± 0.43	1.89 ± 0.31	1.25 ± 0.24	0.42–0.87	2.31
Flexibility (cm)	36.52 ± 5.42	35.84 ± 5.99	37.50 ± 4.53	−6.06–2.75	
CMJ (cm)	36.02 ± 8.03	41.29 ± 5.16	28.37 ± 4.37*	−2.14–4.20	2.70
SJ (cm)	34.82 ± 3.89	35.24 ± 3.89	34.21 ± 4.01	8.99–16.84	
RT (min)	13.78 ± 2.85	15.64 ± 1.85	11.07 ± 1.55*	3.16–5.97	2.67
VO2max (mL/kg/min)	54.45 ± 10.94	59.55 ± 11.46	47.03 ± 3.45 *	5.14–19.89	1.47
HR_LT_ (bpm)	169.67 ± 15.84	173.25 ± 12.19	164.45 ± 19.46	−3.72–21.31	
HR_RC_ (bpm)	187.30 ± 12.62	189.00 ± 12.28	184.82 ± 13.28	−6.05–14.4	
HRmax (bpm)	198.44 ± 9.72	199.19 ± 10.83	197.36 ± 8.21	−6.13–9.78	
_V_RC (km/h)	12.04 ± 1.78	12.90 ± 1.73	10.80 ± 0.93 *	1.04–3.16	1.51
_V_VO2max (km/h)	14.27 ± 1.71	15.38 ± 1.18	12.66 ± 0.83 *	1.93–3.51	2.66
Court Test					
Serve (km/h)	163.19 ± 17.62	174.69 ± 1.92	146.45 ± 8.63 *	19.58–36.89	2.70
Forehand (km/h)	132.96 ± 13.41	139.00 ± 13.69	124.18 ± 6.59 *	6.63–23.00	1.38
Backhand (km/h)	121.85 ± 11.19	127.19 ± 10.45	114.09 ± 7.08 *	5.64–20.56	1.47

Note: CMJ: countermovement jump; SJ: squat jump; RT: running time; VO2max: maximal oxygen uptake; HR_LT_: heart rate at lactate threshold; HR_RC_: heart rate at respiratory compensation; _V_RC: velocity at respiratory compensation; _V_VO2max: velocity at maximal oxygen uptake; * *p* < 0.05 denotes significant differences between male and female tennis players.

**Table 3:** Isokinetic measurements (mean ± SD) of youth male and female tennis players.

Variable	Entire Group (n = 27)	Males (n = 16)	Females (n = 11)	95% CI of the Difference Lower–Upper
Right quadriceps 60 deg/s (Nm/kg)	2.88 ± 0.53	3.08 ± 0.43	2.57 ± 0.53 **	0.13–0.89
Left quadriceps 60 deg/s (Nm/kg)	2.77 ± 0.65	3.09 ± 0.64	2.31 ± 0.30 **	0.41–1.16
Right hamstring 60 deg/s (Nm/kg)	1.92 ± 0.42	2.19 ± 0.24	1.50 ± 0.25 **	0.50–0.90
Left hamstring 60 deg/s (Nm/kg)	1.91 ± 0.46	2.20 ± 0.32	1.48 ± 0.27 **	0.47–0.96
Right quadriceps 300 deg/s (Nm/kg)	1.40 ± 0.31	1.58 ± 0.20	1.13 ± 0.23 **	0.28–0.62
Left quadriceps 300 deg/s (Nm/kg)	1.33 ± 0.28	1.50 ± 0.21	1.08 ± 0.15 **	0.28–0.58
Right hamstring 300 deg/s (Nm/kg)	1.12 ± 0.26	1.28 ± 0.17	0.89 ± 0.19 **	0.25–0.53
Left hamstring 300 deg/s (Nm/kg)	1.19 ± 0.28	1.32 ± 0.25	0.99 ± 0.19 **	0.15–0.51

Note: ** *p* < 0.01 denotes significant differences between male and female tennis players.

**Table 4:** Correlations between anthropometric/physical measurements and strike and serve velocities of young tennis players.

Variable	Serve Velocity (km/h)		Forehand Velocity (km/h)		Backhand Velocity (km/h)	
	Males (n = 16)	Females (n = 11)	Males (n = 16)	Females (n = 11)	Males (n = 16)	Females (n = 11 )
Age (years)	0.39	0.28	0.03	0.39	0.11	0.45
Height (cm)	0.60 *	0.46	0.58 *	0.19	0.30	0.29
Weight (kg)	0.35	0.69*	0.10	0.55	0.017	0.37
Body fat (%)	−0.47	0.30	−0.33	0.41	−0.42	0.12
Flexibility (cm)	0.29	−0.42	−0.09	−0.32	−0.16	0.05
Right quadriceps at 60 degrees/s (Nm/kg)	0.50	0.40	0.21	−0.09	0.22	−0.57
Left quadriceps At 60 degrees/s (Nm/kg)	0.37	0.49	−0.05	0.29	0.11	0.03
Right hamstring at 60 degrees/s (Nm/kg)	0.37	0.18	0.14	−0.46	0.02	0.46
Left hamstring at 60 degrees/s (Nm/kg)	0.36	0.59	0.02	−0.08	0.07	−0.17
RT (min)	0.62 *	0.12	0.43	0.40	0.52 *	0.21
VO2max (mL/kg/min)	0.36	−0.44	0.32	−0.12	0.15	0.09
SJ (cm)	−0.08	0.25	−0.42	0.37	−0.24	0.19
CMJ (cm)	−0.09	0.20	−0.36	0.30	−0.17	0.23
Relative right handgrip strength (kg/BMI)	0.63 **	0.28	0.70 **	0.23	0.62*	−0.01
Relative left handgrip strength (kg/BMI)	0.61 *	0.07	0.65 **	−0.07	0.56 *	−0.18

Note: RT: running time; VO2max: maximal oxygen uptake; SJ: squat jump; CMJ: countermovement jump. * *p* < 0.05; ** *p* < 0.01.

## Data Availability

Data can be obtained by contacting the lead author, K.P.
